# Barriers and facilitators to the management of mental health complications after mild traumatic brain injury

**DOI:** 10.2217/cnc-2020-0022

**Published:** 2021-06-15

**Authors:** Noah D Silverberg, Thalia Otamendi, Amanda Dulai, Ripenjot Rai, Jason Chhina, Anna MacLellan, Pierre-Paul Lizotte

**Affiliations:** 1 Department of Psychology, University of British Columbia, Vancouver, BC, V6T 1Z4, Canada; 2 Rehabilitation Research Program, Vancouver Coastal Health Research Institute, Vancouver, BC, V5Z 2G9, Canada; 3 Department of Family Practice, University of British Columbia, Vancouver, BC, V6T 1Z3, Canada; 4 Department of Family Medicine, Providence Health Care, Vancouver, BC, V6Z 1Y6, Canada

**Keywords:** clinical practice guidelines, family medicine, knowledge translation, mental health, mild traumatic brain injury, primary care, theoretical domains framework

## Abstract

**Background:**

Clinical practice guidelines for mild traumatic brain injury (mTBI) management call on family physicians to proactively screen and initiate treatment for mental health complications, but evidence suggests that this does not happen consistently. The authors aimed to identify physician-perceived barriers and facilitators to early management of mental health complications following mTBI.

**Methods & results:**

Semi-structured interviews based on the Theoretical Domains Framework (TDF) were conducted with 11 family physicians. Interview transcripts were analyzed using directed content analysis. Factors influencing management of mental health post-mTBI were identified along five TDF domains.

**Conclusion:**

Family physicians could benefit from accessible and easily implemented resources to manage post-mTBI mental health conditions, having a better defined role in this process, and formalization of referrals to mental health specialists.

## Lay abstract

Best practice recommendations call for family doctors to proactively screen for and treat mental health problems that might arise after a mild traumatic brain injury (mTBI) (also known as a concussion). However, previous studies show that these recommendations are not followed consistently. The authors aimed to find reasons why this is not happening by interviewing 11 family doctors. The authors found that family doctors are mostly unaware of resources to guide their practice, are unsure about their role in mental health management, and experience difficulties in referring their patients to specialists for mental health care. Actively providing family doctors with knowledge and tools that help them initiate mental health care, as well as a structure for mental health referrals after mild traumatic brain injury, would help doctors apply best practice recommendations.

## Graphical abstract

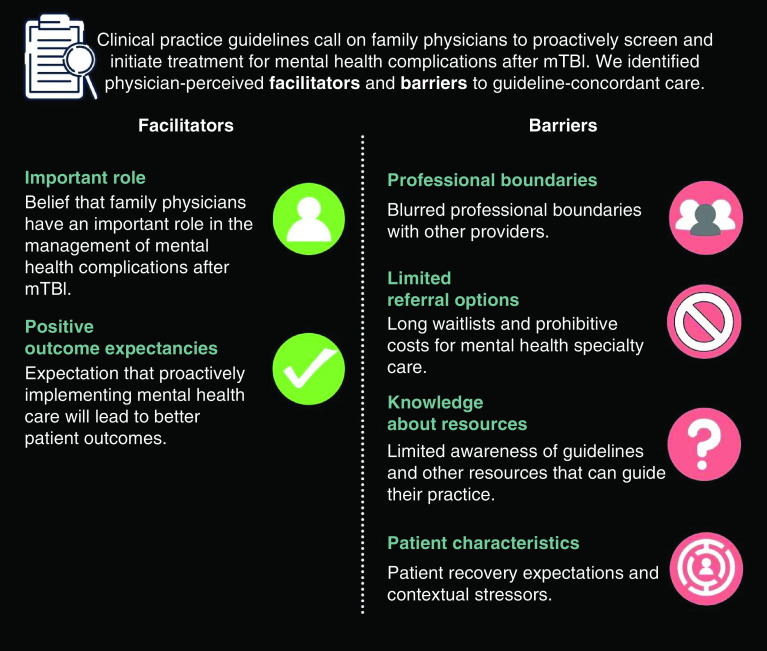

Of the 1–1.5% of the general population who sustain a mild traumatic brain injury (mTBI) every year [1], as many as one in four will develop a mental health disorder such as depression or post-traumatic stress disorder within 6 months of the injury [2,3]. Mental health complications, more than other prognostic factors (e.g., the presence of loss of consciousness), substantially increase the risk of chronic disability after mTBI [2,4–6]. Patients with chronic disability after mTBI accrue substantial costs to society as a result of productivity loss and significant healthcare utilization [7].

Recent clinical practice guidelines for the management of mTBI highlight the importance of screening and initiating treatment for mental health complications in primary care [8,9]; this is based on evidence that mood, anxiety and trauma-related disorders are common and problematic and can be managed with medication and psychotherapy [10–12]. Furthermore, management of these mental health problems may improve other symptoms attributed to mTBI, such as fatigue and concentration problems [8,13]. That said, mental health disorders after mTBI often go undiagnosed and/or untreated, especially in primary care. Less than half of patients who are diagnosed with a mental health disorder after mTBI receive mental health treatment [14–16], indicating a major knowledge–practice gap.

The Theoretical Domains Framework (TDF) [17] has been shown to be effective in identifying barriers and facilitators to clinician behavior change [18], such as adoption of new evidence-based practice guidelines [19,20]. The TDF incorporates a broad spectrum of individual and organizational theories to better understand the motivations of healthcare professionals. The purpose of this qualitative study was to advance the understanding of why best practice guidelines for management of mental health disorders following mTBI are not consistently followed (barriers) and what can be done to improve adherence to the guidelines (facilitators). The TDF was used to guide data acquisition and analysis. The authors focused on family physicians (FPs) because they typically have the most contact with patients over the weeks to months following mTBI [21–23], when mental health complications arise. Clinical practice guidelines for mTBI [8,9] suggest that FPs are best positioned to implement early mental health screening and treatment (e.g., provide supportive counseling, trial an antidepressant medication or refer to a mental health specialist).

## Methods

### Procedure

The authors conducted a qualitative study with semi-structured interviews. The interview guide the authors developed was structured using the TDF and revolved around participants' experiences with management of mTBI, specifically with mental health complications. Example questions are found in Table 1. The more colloquial term ‘concussion' was used in the interviews to refer to mTBI. Interviews occurred either in person at a location of the participant's choice or over the telephone using a secure web-based teleconference service (OpenVoice). Participants were sent a written consent form and asked to return a signed copy before the interview session. The session began by verbally reconfirming eligibility and consent, asking the participants standardized questions about their demographic and clinical practice characteristics, and then proceeded with the TDF-based interview. Interviews were completed by family medicine residents (one female, two male) who were members of the research team. Training was provided for the interviewers through mock interviews by personnel with experience in qualitative interviews. Interviewers' initial audio recordings were also reviewed by members of the team with qualitative interviewing experience, and feedback was given regarding strategies for developing rapport with the participants before starting the formal interview, facilitating a conversational discussion, and using follow-up probes for participants to elaborate on their initial answers. The interviews occurred between February and April 2019 and were audio recorded and transcribed verbatim by a professional transcription service. Interviews lasted between 19 and 49 min (mean = 27; standard deviation = 8).

**Table 1.** Theoretical Domains Framework-based interview questions.

Theoretical domains	Example questions
Knowledge	How common do you think mental health problems are after a concussion? How much do they influence recovery from concussion?
	How strong is the scientific evidence for mental health treatments in patients with concussions? How helpful is the evidence for guiding you to take one action versus another?
Skill	Which skills are most important for a family physician to have in managing post-concussion mental health problems?
Social/professional role	What role should the family physician play in managing mental health problems after concussion?
Beliefs about capabilities	How confident do you feel in your ability to identify when your patient has significant depression or anxiety after concussion?
	How confident do you feel in your ability to initiate treatment for depression or anxiety after concussion?
Beliefs about consequences	What difference does it make to a patient's long-term outcome from concussion when a family physician takes action to address mental health problems?
Memory, attention and decision process	To what extent do you remember to screen for mental health issues after concussion?
Environmental context and resources	To what extent do you use any clinical practice guidelines or expert consensus statements to help you manage a patient with concussion? Which one(s)? How do you use them? How are they helpful (or not)?
	How available is counseling or psychological treatment for your patients with concussion?
	What other resources do you use to help you manage a patient with concussion who is presenting with mental health problems?
Social influences	To what extent do patients with concussion expect you to ask about their mental health or want to talk about their mental health problems?
Emotion	To what extent are there certain patients with concussion that make you more concerned or worried?
Behavioral regulation	What might help you to consistently screen patients for mental health problems after concussion?
Goals	What do you hope to achieve by screening for mental health problems after concussion?
Reinforcement	What kind of experiences have you had in the past that influence your management of post-concussion mental health problems today?
Intention	To what extent do you intend to be more attentive to potential mental health problems after concussion?
Optimism	To what extent do you expect that screening and initiating treatment for mental health problems after concussion will lead to better patient outcomes?

### Participants

Participants were eligible for the study if they were fully licensed FPs, had a practice located within the Greater Vancouver area, had been in independent practice for at least 1 year, and devoted at least 50% of their time to an outpatient general practice. Participants were selected using a purposive sampling strategy with the aid of the College of Physicians and Surgeons of British Columbia's public registry to identify and recruit FPs who were eligible for the study. The authors identified a pseudorandom selection of 167 potential participants. These physicians, who appeared to meet the study eligibility criteria (based on information available in the College of Physicians and Surgeons of British Columbia's website directory), were sent a letter of initial contact inviting them to contact the authors' team. Potential participants who did not contact the authors' team within 10 days were telephoned by a research team member and invited to participate in the study. Purposive recruitment was supplemented by snowball sampling (outreach to potential participants through referrals from FP participants) and convenience sampling (referrals from study team members). The authors were unable to collect data on reasons for nonparticipation. No FPs replied to the letter of invitation to express interest but not proceed with enrollment. Of the FPs identified through snowball sampling, a minority (n = 4) expressed interest but did not respond to scheduling requests.

### Data analysis

Interview transcripts were imported into the qualitative data analysis software NVivo. Recommendations for the application of the TDF to identify implementation problems [18] were followed using directed content analysis [24] to deductively code the participants' accounts along the domains of the TDF. In-depth data familiarization was achieved by reading the entire data set several times and highlighting all meaning units (manifest and latent) that were deemed relevant to barriers and facilitators to the identification and initiation of treatment for post-mTBI mental health complications. To increase reliability, two researchers (T Otamendi and A MacLellan) independently engaged in the process and met several times to refine coding procedures and ensure consistency for the analyses. For example, they conferred after independently coding the first four interviews, which resulted in an agreement of 84% [18,25]. The researchers resolved any differences through discussion, further analysis and consensus. The remaining differences were discussed with an additional member of the research team (ND Silverberg), who assisted with the final decisions. Through this approach, relevant barriers and facilitators within the TDF domains were identified by their prominence in the transcripts (i.e., frequency and presence of strong beliefs) [18]. Participants' direct quotations were used to supplement the findings. The consolidated criteria for reporting qualitative research [26] were used to ensure that relevant aspects of qualitative research were reported. A copy of the checklist is provided in Supplementary File 1.

## Results

The final sample consisted of 11 FPs, with characteristics summarized in Table 2. An administrative error resulted in missing demographic data for one participant. Participants had regular but infrequent (~1/month) experience caring for patients with mTBI. Using directed content analysis, the authors identified the following five prevalent TDF domains: professional role and identity, environmental context and resources, knowledge, beliefs and consequences, and beliefs about capabilities. Within these domains, the barriers and facilitators identified were: perceived role in post-mTBI mental health care, positive outcome expectancies, challenges within the healthcare system, need for consolidated and concise material resources, knowledge of condition based on clinical experience, barriers to patient engagement, and diagnostic ambiguity.

**Table 2.** Demographic, professional and practice characteristics.

Demographics	n^†^ (%)
Male sex	8 (80)
Special competence designation	
Emergency medicine	2 (20)
Addictions	1 (10)
None	7 (70)
Urban/rural practice	
Urban	6 (60)
**Additional practice details**	**Median (range)**
Number of years practicing post-residency	12 (3–40)
Estimated number of mTBI patients seen in past year	12.5 (6–50)
Estimated number of mTBI patients seen in past month	2.5 (0–10)

† n = 10 because demographic data were missing for one participant as a result of administrative error.

mTBI: Mild traumatic brain injury.

### Professional role & identity

#### Perceived role in post-mTBI mental health care

FPs firmly believed that they should have an integral role in managing mTBI, including associated mental health complications:

*I think the role as family physician is crucial because we are the ones with long-term, longitudinal relationships with these patients, and so we see them month after month, and they have to feel supported by their family doctor month after month.* (Participant 10)

That said, the nature of the role FPs should play was less defined. Whereas some participants perceived themselves to be competent to screen for and diagnose anxiety and depression in mTBI, others were less certain that they had the knowledge and resources to treat these conditions. In general, FPs perceived blurred professional boundaries in the treatment of post-mTBI mental health conditions and preferred to have the option of referring to specialized or allied healthcare practitioners:

*I think the details and intricacies of therapy might be the realm of another profession. For example, more in-depth counseling that might take an hour visit might be better done by a counselor or psychologist. Then therapies, like looking at return to school or work, might be better done by an occupational therapist*. (Participant 2)

Because of their broad role in management, FPs often felt obligated to initiate treatment for mental health complications following mTBI but experienced limitations, such as lack of training and/or lack of time. FPs expressed that better defined roles could allow them to focus on what they consider the most appropriate tasks for an FP: screening for mental health problems, coordinating care, and providing education to patients about the potential involvement of mental health complications in mTBI recovery.

### Beliefs about consequences

#### Positive outcome expectancies

FPs were motivated to screen for mental health problems because of their expectation that proactively implementing care would lead to better patient outcomes:

*I would expect that both screening, identifying and potentially treating mental health issues in the context of concussion would potentially help them to get better faster, both from the mental health perspective and, in theory, from the concussion elements*. (Participant 7)

Based on their clinical experience, FPs also perceived that the earlier the problems were addressed, the more manageable they would be:

*I think that can sometimes be the difference between helping someone return to work in a timely fashion versus a prolonged disability – and sometimes a patient who is unable to return to work. I think, as a family doctor, you have an ability to empower your patient and reassure them. Doing that early on in a first or second visit of a concussion is instrumental to recovery.* (Participant 2)

FPs perceived that early intervention established a positive therapeutic alliance and treatment plan that helped patients avoid a potentially stressful process:

*People get stuck in a path of thinking, ‘No one knows what's going on, and I don't know what's going on, and this is really depressing,’ whereas if you can say, ‘Yeah, this feels crappy, but we know exactly what it is. It's part of the brain that's damaged, and look at these things it's doing to you, but the good part is this gets better.’ So, I think that helps their recovery.* (Participant 5)

Anticipating positive outcomes prompted FPs to initiate mental health care as best they could.

### Environmental context & resources

#### Limited referral options for specialized care

The primary environmental barrier was a perception of limited access to specialized mental health care in the public healthcare system. FPs mentioned that long wait lists and prohibitive costs often dissuaded patients from seeking treatment with counselors, psychologists, and psychiatrists:

*I think the current system is that the family physician does all of it. I don't think that there are very many easily and timely accessible options for people who are suffering from mental health problems following a concussion.* (Participant 11)

For those patients who can afford it or have extended health coverage, FPs talked about private care being an option. However, this alternative was also perceived as not readily available:

*I just can't get private psychiatrists to see any patients about anything. There are just not enough of them*. (Participant 10)

Necessitating additional support but struggling to receive it, FPs felt that the entirety of care resided with them, and they felt obligated to treat post-mTBI mental health as best they could (typically by offering antidepressant medication) while continuing to search for specialist referral options:

*My referral was rejected. They said that it's not the type of conditions they see. I guess they see mostly schizophrenia and those types of problems, but this is treatment-refractory depression. So the strategy is, I guess, keep trying . . . keep referring to different places.* (Participant 1)

In all, FPs perceived that a lack of structured support in post-mTBI mental health care was a significant obstacle to providing optimal care.

#### Need for consolidated & concise material resources

During appointments with mTBI patients, FPs perceived to have helpful material resources to separately assess mental health and to guide a return to activity as symptoms allowed. Specific resources mentioned by name included the Patient Health Questionnaire-9 and the Generalized Anxiety Disorder-7 to screen for depression and anxiety, the Sport Assessment Concussion Tool-5 to track mTBI symptoms, and Parachute Canada's Return to Play/Activity guidelines to guide patients in their return to sports and other activities. However, FPs were not aware of consolidated tools to facilitate intervention for mental health complications after mTBI:

*Most of the concussion-related guidelines I use are all about return to work, return to play, but most of those don't . . . they don't typically look at mood disorders.* (Participant 8)

Therefore, FPs described using general mental health screening tools for mTBI patients but were unsure if this was appropriate and if treatment should be modified for these patients:

*I think similar guidelines to treatment of anxiety or depression following concussion would be a benefit because I have to admit, as of right now, if I had a patient who was suffering from anxiety or depression post-concussive, I would treat it as I would any other patient that has symptoms of depression and anxiety*. (Participant 3)

Finally, FPs perceived some of these existing materials to be long and not easily implementable in an FP office practice:

*The problem is that these are long guidelines that are not distilled down for primary care. If they were distilled down for primary care and had templates to be practically used in practice, it would be very useful*. (Participant 4)

FPs mentioned their preference for brief (one page) print or digital materials that consolidated mTBI and mental health information and were readily accessible to themselves and their patients.

### Knowledge

#### Knowledge based on clinical experience

When asked how common they perceived post-mTBI mental health complications to be, FPs answered that they were quite common, providing estimates that ranged between 25 and 50% of mTBI patients. This understanding was based mostly on their clinical experience:

*I mean, I've had the experience of seeing hundreds of patients and watching them recover or not recover, sometimes over years.* (Participant 8)

FPs acknowledged that they were not up to date on current scientific literature and therefore could not comment on the supporting evidence of post-mTBI mental health management:

*I have to admit, I'm not up on the latest research. So, as far as scientific evidence, I don't know if I can speak to that. I don't know the strength of the evidence*. (Participant 3)

Most FPs seemed unaware that there were evidence-based guidelines available to help in the management of post-mTBI mental health. They expressed that if this consolidated source of knowledge existed, it should be disseminated and distilled down for primary care. Without this, they expressed a lack of confidence in having to develop their own care strategies:

*The challenge is that there's no education from the system or from healthcare providers as to how the anxiety or depression symptoms differ from typical major depressive disorder or anxiety disorder.* (Participant 4)

FPs talked about reluctantly having to rely on their intuition rather than existing guidelines to guide practice.

### Beliefs about capabilities

#### Barriers to patient engagement

FPs identified certain patient characteristics (e.g., premorbid personality pathology, lack of awareness of potential post-mTBI mental health complications) and contextual stressors (e.g., medicolegal involvement) that may limit the effectiveness of mental health treatment in the primary care setting:

*I think of patients I know who are just barely getting by, struggling with family issues prior to a concussion, and then have a concussion kind of just tip them over the edge. When I see those patients, I feel like a treatment plan has to be a bit more comprehensive and sometimes beyond what I can do just in the office.* (Participant 2)

FPs also suggested that a patient's lack of awareness that mental health is involved in their recovery could pose a barrier to accepting treatment:

*I don't think they really have an expectation of that. I don't think most of them are really aware that concussion causes that. Most patients know that concussion causes headache and dizziness and inability to work and things like that, but I don't think most patients really understand that it can cause depression, anxiety, long-term mental health problems.* (Participant 8)

In some cases, this lack of awareness became more troublesome when patients resisted the idea that mental health could be involved in their recovery. Although FPs did not explicitly mention it, this presumably added yet another step to the treatment process, as patients would have to be persuaded of the involvement of mental health in recovery:

*They are primarily focused on their recovery and what has physically happened [. . .] in general. They want to attribute their symptoms to their concussion and not necessarily to a mental health disorder.* (Participant 7)

Certain patient characteristics and contextual situations were perceived to diminish the already limited confidence FPs had in their capabilities to address these complications.

#### Diagnostic ambiguity

Although FPs were aware that mTBI and mental health disorders commonly co-occur, certain characteristics made them difficult to assess, differentiate and manage. For example, an important source of confusion seemed to be the nonspecific nature of mTBI and mental health symptoms as well as the overlap between them:

*Again, because lots of the cognitive effects of concussions have challenges that can sometimes mimic or look similar to depression, it's oftentimes very difficult. Unless they have a fairly established baseline of mental illness, it makes the diagnostics quite challenging.* (Participant 7)

They perceived that diagnostic clarity was even more challenging to achieve when certain patients were motivated to downplay or exaggerate symptoms, introducing uncertainty about the results of their assessments:

*I guess just really trying not to miss people who might be having mental health issues who aren't as forthcoming about it*. (Participant 9)

Furthermore, patients' focus on physical symptoms during consultations as well as the potential late onset of mental health symptoms made it challenging to determine which patients to proactively intervene with:

*[Mental health symptoms] kind of fester over time, as a focus on physical symptoms may be taking place. You might find yourself, months down the road, where you have a full-blown major depressive episode that now has become a big problem and is preventing the person returning to work or school*. (Participant 2)

In all, ambiguity surrounding mTBI and mental health diagnoses and treatment was perceived by the FPs as a challenge to their own capability to correctly detect and manage these injury complications. They expressed concern that mental health complications could go undiagnosed in the early stages of recovery and manifest as larger problems in the long-term.

## Discussion

A deductive qualitative analysis rooted in the TDF led the authors to identify multiple barriers and facilitators to implementing early management of mental health complications following mTBI in primary care. FPs generally perceived themselves to have a prominent role in post-mTBI care (professional role and identity), specifically screening for mental health problems, providing education about mTBI, and coordinating care. Additionally, FPs believed that managing post-mTBI complications facilitated better patient outcomes (beliefs about consequences), motivating them to intervene. These facilitators are likely offset by significant barriers, including not having a clearly defined role in the treatment of mental health vis-à-vis other mental health professionals (professional role and identity). FPs were generally not aware of evidence-based guidelines or tools to guide their practice (knowledge). As such, they hesitantly relied on general mental health screening tools and attempted to refer to specialized mental health practitioners for support, but encountered difficulties in finding accessible referral streams (environmental context and resources). Finally, FPs described the process of diagnosing mental health conditions after mTBI as challenging, explaining that certain patient factors (e.g., premorbid personality) and contextual factors (e.g., injury compensation seeking), as well as the subjective and nonspecific nature of symptoms, decreased their confidence in detecting and treating mental health complications (beliefs about capabilities).

FPs' limited familiarity with evidence-based and expert consensus recommendations for identifying and managing mental health problems after mTBI is consistent with research reporting low uptake of clinical practice guidelines in general [27–30], including mTBI-specific guidelines [31–35]. Reasons for the limited incorporation of these tools in primary practice may be multifaceted. Individual-level barriers are regularly reported in the literature [35]. For example, the lack of awareness of the existence of clinical care guidelines reported by the FPs in this study is a common finding [36–38]. Previous research has reported that practitioners' comfort using ‘knowledge in practice' to guide clinical judgment [39,40] and resistance to the ‘cookie-cutter’ approach to care [40,41] may serve as obstacles to seeking published guidelines. FPs' perception of having an integral role in post-mTBI mental health care and positive expectations of proactive management, together with their self-awareness of knowledge gaps, motivated them to pursue learning opportunities regarding mTBI and mental health. For instance, they sought confirmation that it was reasonable to use standard mental health screening measures (e.g., Patient Health Questionnaire-9 for depression) in patients with mTBI, which practice guidelines recommend [10] based on evidence that such measures perform similarly in mTBI [42–45]. Limited beliefs in their abilities may stem from FPs' relatively infrequent exposure to mTBI patients. Previous research has reported that the majority of FPs see less than ten mTBI patients per year [46], a figure similar to that seen in the present study. This might prevent them from maintaining knowledge of best practice and confidence in managing these patients [19,47]. This study suggests that FPs are motivated and open to incorporating evidence-based knowledge into their management of post-mTBI mental health care but experience barriers to accessing such information.

Passive dissemination of guidelines (i.e., through publication) rarely leads to sustained changes in clinical practice [38,48]. This may be due to an overload of information that is not readily applied in practice and is consistent with the FPs' perceptions that existing knowledge is likely not distilled down for primary care. Thus, this study supports recommendations that mTBI guidelines should be more ‘implementable' (e.g., adaptable, usable, easily communicated) [49] and disseminated through active knowledge transfer strategies, such as tailored recommendations, decision support tools, or interactive education sessions [38,50,51].

Guideline implementation tools alone may not be enough to achieve optimal care for post-mTBI mental health problems. In accordance with guidelines [8,9], FPs will need to be able to refer patients with mTBI to mental health specialists in situations of diagnostic uncertainty, complex symptom presentations, or if initial treatment is not effective. FPs reported encountering limited options and long wait lists in the public healthcare system, as well as prohibitive costs for patients and referral rejections in the private healthcare system. Therefore, organizational interventions may be necessary to implement structured referral processes and involve specialists in the dissemination of best care practices [52].

## Limitations

The number of participants interviewed was limited pragmatically by the training program-dictated timeline for the involvement of family medicine residents on the authors' research team. It is possible that interviewing additional FPs would have enhanced the richness of the data and allowed conflicting opinions to emerge, but the sample size was enough to achieve thematic saturation, which was reached by the eighth interview. Therefore, the authors' sample size seemed adequate for the relatively homogeneous population and study objectives. Although the interviewers of the research team received training, their limited experience in qualitative interviewing may have also impacted the depth of the data collected. This weakness is weighed against the strength of having family medicine residents do the interviewing, which may have helped with FP participant engagement. Selection bias was likely, due to the sampling strategies used and the volunteer nature of the study, where FPs who agreed to participate may have had an above average awareness of and interest in the topic of post-mTBI mental health. Using the TDF to inform the interview guide allowed for a theoretical basis to the study but may also have biased answers toward those associated with TDF domains rather than ones naturally reported. Finally, the authors did not observe marked differences between male and female participants because of the sample size, which did not allow adequate exploration of potential sex and gender differences. This is an important avenue for future research.

## Conclusion

To our knowledge, this is the first study to investigate potential barriers and facilitators to implementing clinical practice guidelines regarding screening and initiating treatment for mental health complications after mTBI in primary care. This study can inform future interventions to improve implementation. The results suggest that addressing FP knowledge of evidence-based post-mTBI mental health management guidelines, introducing tailored implementation tools that highlight specific actions the FP could consider, and building accessible referral pathways to mental health specialists may be most impactful. Timely treatment for mental health complications following mTBI would improve patient outcomes from mTBI.

## Future perspective

Mental health conditions are increasingly being recognized as barriers to recovery from mTBI. Considering that evidence-based recommendations for post-mTBI mental health conditions are not consistently applied, we expect that implementation efforts will address this knowledge–care gap in the next decade. The findings from this study can inform such efforts. Along with other pillars of evidence-based mTBI management, such as encouraging a prompt return to activity and multidisciplinary treatment, we expect that the implementation of mental healthcare guidelines will decrease the burden of chronic disability after mTBI.

**Summary points**

- Clinical practice guidelines for mild traumatic brain injury management recommend screening and initiating treatment for mental health complications in primary care, but passive dissemination of guidelines has not led to sustained changes in clinical practice.
- Family physicians experience barriers to guideline implementation, such as lack of role clarity in managing post-mild traumatic brain injury mental health care, lack of awareness of existing knowledge and tools to guide their practice, and difficulties in finding accessible referral streams to mental health specialists.
- Family physicians could benefit from active guideline implementation strategies, such as providing succinct knowledge and accessible tools for post-mild traumatic brain injury mental health management, tailored treatment recommendations, and formalization of referral processes to mental health specialists.
